# Genotypic Tannin Levels in *Populus tremula* Impact the Way Nitrogen Enrichment Affects Growth and Allocation Responses for Some Traits and Not for Others

**DOI:** 10.1371/journal.pone.0140971

**Published:** 2015-10-21

**Authors:** Franziska Bandau, Vicki Huizu Guo Decker, Michael J. Gundale, Benedicte Riber Albrectsen

**Affiliations:** 1 Department of Plant Physiology, Umeå Plant Science Centre, Umeå University, SE 90183 Umeå, Sweden; 2 Department of Forest Ecology and Management, Swedish University of Agricultural Sciences, SE 90183 Umeå, Sweden; 3 Department of Plant and Environmental Sciences, University of Copenhagen, Thorvaldsensvej 40, DK 1871 Frederiksberg C, Denmark; Shandong University, CHINA

## Abstract

Plant intraspecific variability has been proposed as a key mechanism by which plants adapt to environmental change. In boreal forests where nitrogen availability is strongly limited, nitrogen addition happens indirectly through atmospheric N deposition and directly through industrial forest fertilization. These anthropogenic inputs of N have numerous environmental consequences, including shifts in plant species composition and reductions in plant species diversity. However, we know less about how genetic differences within plant populations determine how species respond to eutrophication in boreal forests. According to plant defense theories, nitrogen addition will cause plants to shift carbon allocation more towards growth and less to chemical defense, potentially enhancing vulnerability to antagonists. Aspens are keystone species in boreal forests that produce condensed tannins to serve as chemical defense. We conducted an experiment using ten *Populus tremula* genotypes from the Swedish Aspen Collection that express extreme levels of baseline investment into foliar condensed tannins. We investigated whether investment into growth and phenolic defense compounds in young plants varied in response to two nitrogen addition levels, corresponding to atmospheric N deposition and industrial forest fertilization. Nitrogen addition generally caused growth to increase, and tannin levels to decrease; however, individualistic responses among genotypes were found for height growth, biomass of specific tissues, root:shoot ratios, and tissue lignin and N concentrations. A genotype’s baseline ability to produce and store condensed tannins also influenced plant responses to N, although this effect was relatively minor. High-tannin genotypes tended to grow less biomass under low nitrogen levels and more at the highest fertilization level. Thus, the ability in aspen to produce foliar tannins is likely associated with a steeper reaction norm of growth responses, which suggests a higher plasticity to nitrogen addition, and potentially an advantage when adapting to higher concentrations of soil nitrogen.

## Introduction

Forest ecosystems in the far northern latitudes (i.e. boreal forests) are strongly limited by nitrogen (N) availability [1, 2], and human activities greatly influence the inputs of N into these ecosystems [3, 4]. Fossil fuel combustion, fertilizer production, and agricultural intensification during the past century have led to a 3- to 5-fold increase in the global emissions of reactive nitrogen, leading to elevated levels of atmospheric N deposition globally, including in the boreal region [4, 5]. Additionally, because N availability strongly limits productivity in boreal forests, forest managers are increasingly applying fertilizers to enhance forest productivity [6]. These anthropogenic inputs of N into N-limited boreal environments are known to have numerous environmental consequences, including shifts in plant species composition and reductions in plant species diversity [7, 8]. In contrast to well described community level changes, knowledge about how genetic differences within plant populations influence responses to eutrophication in boreal forests is scarce [9, 10]. If different genotypes within a species show variable responses to anthropogenic N enrichment, the genetic diversity of a population may serve as a key mechanism that promotes the stability of species within communities in response to anthropogenic change [11, 12].

One factor that could help promote the stability of a given species in response to environmental eutrophication is when a population contains a diversity of individuals with contrasting growth and defense qualities [13–16]. Several hypotheses have been proposed to explain how plants optimally grow or defend themselves in response to variation in nutrient availability [17]. Most of these hypotheses assume that the production of defense compounds comes at a negative cost for growth. The growth differentiation balance hypothesis (GDBH, [18]) proposes that protective metabolites and structures are prioritized by plants in low fertility environments, while growth is emphasized as soils become more nutrient rich. The protein competition model (PCM, [19]), predicts a trade-off between plant growth and defense, because the amino acid phenylalanine is a common precursor in the synthesis of both proteins and phenolic defense compounds. According to the PCM model, N uptake determines whether photosynthate is directed towards growth via protein synthesis (i.e. N demanding) or the production of phenolic compounds (that do not contain N). While trade-offs between growth and defense have been evaluated among and within species [11, 20–24], less attention has been paid to allocation differences for genotypes within a species that express extreme differences in their baseline investment into defense chemicals (but see [25]). Such variation in genotype response may be key in understanding how sensitive or resilient individual species are to environmental change.

One particular class of foliar defense compounds, condensed tannins, have been shown for several species to vary substantially among different genotypes [24, 26–28]. Condensed tannins are carbon-based polyphenolic compounds that defend plant foliage through their astringency, and by making plant biomass less digestible due to their complexation with proteins [29]. Some plants invest a considerable proportion of their mass into condensed tannins. Condensed tannin levels have been shown to have a large degree of natural variation among individuals of the same tree species in forests (e.g. from 12–27% dry weight (DW) [26] or from 14–27% DW [30] in *Populus tremuloides*, from 6–24% and 10–22% DW during July in *Quercus alba* and in *Quercus velutina*, respectively [31]). While it is known that condensed tannin concentrations can show substantial plastic responses to herbivory (i.e. induced defense) [29, 32], or soil nutrient concentrations [11, 33], it is also recognized that genetic differences among individuals contribute to this variation [11, 14, 34]. For example, Lindroth and Hwang [26] showed that condensed tannin concentrations varied 2.1-fold between contrasting genotypes of *Populus tremuloides*, whereas Haikio et al. [27] found that condensed tannin concentrations in two hybrid aspen clones (*P*. *tremula x P*. *tremuloides*) varied 3.5-fold. However, to date it remains poorly understood how plant genotypes with contrasting baseline condensed tannin concentrations influence their growth response to levels of environmental eutrophication that are typical in semi-natural forest environments, such as occur through atmospheric N deposition or industrial forest fertilization programs.

In this study we focus on a model tree species, *Populus tremula* (i.e. European aspen), to investigate whether different genotypes respond differently to nutrient enrichment, and whether underlying differences in their baseline tannin production affect the expected trade-off between growth and defense. European aspen and its close North American relative *P*. *tremuloides* have a circumpolar distribution in the northern hemisphere, and serve as keystone species in part by hosting a highly diverse community of arthropods [35–37], and fungi [35, 38], and by serving as a preferred food for several larger herbivores [39–41]. *Populus* exhibits a high level of outcrossing that results in a high intraspecific genetic diversity [42–43], and possesses highly heritable defense and growth traits [36], making it an ideal study object. With genome information from *Populus trichocarpa*, which was the first tree genome to be sequenced [44], intensive molecular background work and phenotyping has been carried out. In *Populus tremula* intraspecific variation has been studied for several traits including genetic structure [45], architecture [46], phenology [47], growth [36], foliar chemistry [48, 49], as well as herbivore and pathogen susceptibility [36, 50]. Molecular advances have also led to hopes of a better understanding on the regulation of tannin biosynthesis [29]. Using young replicates of 10 *Populus tremula* genotypes that vary greatly in their baseline production of condensed tannins, we addressed the following questions to understand how this species may respond to atmospheric N deposition in the boreal region, as well as to rates of N application increasingly used by the forest industry. First, we wanted to confirm that nitrogen addition and genotype will affect plant growth, allocation of biomass among tissues, and tissue chemistry; and that *Populus tremula* genotypes will respond to N enrichment differently, as previously demonstrated for *Populus tremuloides* (e.g. [23]). Secondly, we asked, whether the intrinsic ability of a genotype to produce tannins would affect allocation towards growth and defense; alone and in interaction with nitrogen enrichment. Specifically, we predicted that genotypes exhibiting high intrinsic tannin levels would be less responsive to N addition, because tannin production requires substantial amounts of carbon (C), thus making it unavailable for other demands like growth. Answering these questions in combination will provide a rare insight into how a tree species may respond to anthropogenic N enrichment at the intraspecific level.

## Material and Methods

### Plant material and experimental design

**Fig 1:**
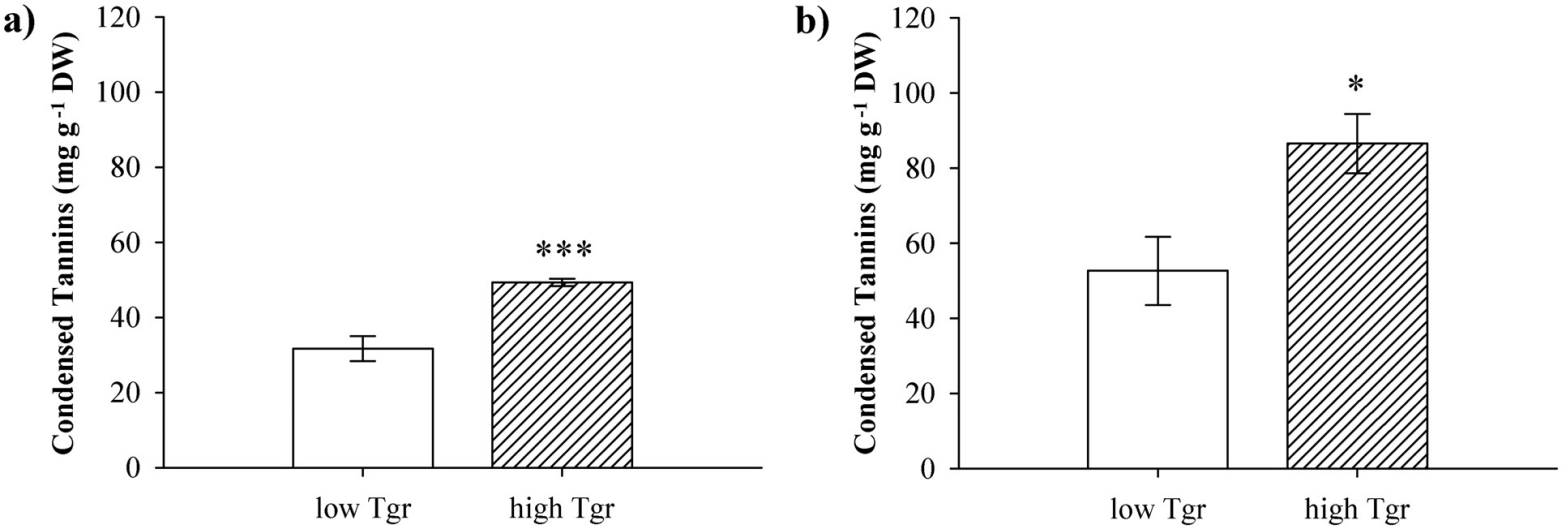
Tannin concentrations in aspens with low and high intrinsic tannin production. Mean condensed tannin concentrations (± SE) in foliage of aspen (*Populus tremula*) with low (white bar) and high (hatched bar) intrinsic tannin production a) from trees of the SwAsp collection sampled during 2008 and 2009, and b) in trees included in this experiment (2011) grown under ambient nitrogen conditions. Please see methods for details. Indication of significance (*** P<0.001, * P<0.05) from an independent-sample t-test testing for differences between tannin groups is given above the hatched bar.

Our study utilized ten European aspen (*Populus tremula*) genotypes (GTs) that exhibited a wide range of variability in their expression of foliar condensed tannins. The GTs originated from the Swedish Aspen Collection (SwAsp), a collection of 116 GTs obtained from throughout Sweden, and planted in two common gardens 1200 km apart [47]. Using the acid-butanol method (described in detail below) (i.e. [36]), we assessed foliar condensed tannin concentrations during 2008 and 2009 for the GTs planted in the common gardens (S1 Fig). Based on this common garden data, we then selected five genotypes that expressed ***low*** levels (SwAsp ID 18, 23, 50, 60, and 115), and five genotypes that had ***high*** levels of foliar condensed tannins (SwAsp ID 5, 26, 51, 65, and 72). Tannin group differences (low vs. high-tannin) were confirmed by an independent sample t-test (t = 5.06, P < 0.001, Fig 1a). The common gardens were established on the land of The Forestry Research Institute of Sweden in Sävar and in Ekebo. No specific permission was required, and the field study did not involve endangered or protected species.

The selected GTs were propagated from in-vitro tissue culture, starting in January 2011. In mid May 2011, 30 clones of each genotype (= 300 individuals) were potted into 5 l pots in a mixture of sand, peat, and loam (51:48:1). The selected plants were chosen to assure uniformity in size (8 cm) and vigor. The plants were kept in the greenhouse (SLU, Umeå, Sweden) at 60% relative humidity, with day and night temperatures of 20°C and 15°C, respectively, and under a 16:8 hour light:darkness regime. The noon light intensity within the greenhouse ranged between 270 and 350 μmol m^-2^ s^-1^ photosynthetically active radiation. Side branches were trimmed within the first four weeks after planting to ensure comparative growth. Fans were used to agitate the plants to promote stem lignification.

In early July, we measured stem height and diameter (1.5 cm above the soil surface) of all plants. We then used stratified random sampling to select three individuals per GT that were representative for the range of growth expressed within each GT. The selected plants were harvested, and their dry mass was assessed. Dry mass, height and diameter measurements were then used to estimate initial biomass of the experimental plants by the use of linear regression equations based on the relationship of size (height*stem diameter^2^) to dry mass as described in [51]. Regression equations were derived for each tannin group separately. We then selected 18 individuals of each GT, which were brought to a wind-sheltered out-door area exposed to ambient sun and precipitation. To avoid initial sun damage, a thin mousseline screen was placed above the plants for the first ten days. The plants were arranged in three blocks with six individuals of each GT per block. Within each block plants were randomly assigned to one of three N treatments: 0 kg N ha^-1^ corresponding to ambient N conditions, 15 kg N ha^-1^ equivalent to high level atmospheric N deposition in the boreal region [5], and 150 kg N ha^-1^ corresponding to industrial forest fertilization levels in Sweden. The treatments were applied on a pot surface area basis. During each of three applications 0, 46 or 460 mg NH_4_NO_3,_ respectively, were added. The fertilizer was dissolved in de-ionized water, and applied on July 7^th^, 21^st^, and August 4^th^. The experiment ran for seven and a half weeks, during which plants were regularly watered, and manually cleared of insect herbivores. Any aphids that were found were locally eradicated with insect soap as needed, and in early August all plants were treated with the fungicide Tilt^®^ 250 EC (Makhteshim-Agan, Leusden, The Netherlands; concentration: 1ml 1l^-1^ H_2_O) to avoid rust outbreak.

### Plant harvest and destructive measurements

On August 23^rd^ 2011, plant height and stem diameter were measured, followed by destructive harvest of each plant. The five youngest, undamaged, fully expanded leaves of each plant were harvested for leaf chemical analyses. They were immediately freeze-dried after harvest, weighed and stored at -20°C. Leaves, stems, and roots were then harvested, separately. Total leaf area of each plant was assessed with a leaf area meter (LI-3000C and LI-3050C, Li-Cor^®^, Lincoln, NE, USA), and the number of leaves per tree was counted. Roots were washed, and all tissues were dried at 60°C until constant weight. Dry weight was assessed for each type of tissue, separately. The different plant tissues were coarse-ground on a Wiley^®^ mill (mesh size #10; Thomas Scientific, Swedesboro, NJ, USA). Some of the coarse-ground material was set aside for lignin analysis (all tissues), whereas the remaining material was further ground to a powder using a ball mill (Retsch^®^ MM 400, Retsch^®^, Haan, Germany). Fine-ground material was used for chemical analysis of condensed tannin (mature leaves only), C and N (all tissues).

### Chemical analyses

Foliar condensed tannin concentrations were assessed using the acid-butanol method [52]. In short, 20.0 ± 2.0 mg leaf powder (exact weight was recorded) was extracted with 800 μl of an acetone solution (70% acetone, 30% Milli-Q water, with 10 mM ascorbic acid), vortexed, sonicated for 4 minutes in an ice water bath, and centrifuged for 5 min at 3500 rpm. Extracts were then reacted with an iron and an acid-butanol reagent, and absorbance at a wavelength of 550 nm was measured using a spectrophotometer (Hitachi U-5100 UV/VIS, Hitachi High-Technologies, Tokyo, Japan). As tannin standard we used procyanidin B2 (C_30_H_26_O_12_, Sigma-Aldrich^®^, St. Louis, MO, USA).

Carbon and N concentrations of all tissues were assessed using dry combustion (LECO TruSpec CN Furnace, LECO Corporation, Lakeview, MI, USA). Lignin was measured using the acid-detergent fibre-sulphuric acid procedure [53]. Carbon, N and lignin analyses were performed at the Soil, Water and Plant Testing Laboratory at Colorado State University.

### Calculation of response variables

From direct measurements, we further calculated a variety of growth and biomass allocation responses, as well as tissue chemistry variables, including average leaf area, relative growth rate for height (RGR), leaf biomass, the root:shoot ratio, total biomass, daily biomass increment, whole plant C and N concentrations, and C:N ratios. Average leaf area represented the ratio of total leaf area, and leaf number. Relative growth rate for height (RGR) was calculated by dividing the difference between final height and initial height by the number of days that had passed between the two height measurements. Leaf biomass was calculated by summing the mass of the freeze-dried leaves harvested for chemical analysis and the mass of the oven-dried leaves. The root:shoot ratio was obtained by dividing root biomass by the sum of leaf and stem biomass. Total biomass was the sum of leaf, stem, and root biomass. Daily biomass increment was calculated by dividing the difference between final total biomass and the initial biomass estimate by the number of days between the two biomass measurements. Whole plant C and N concentrations were calculated by determining C and N contents in the different tissues first ((tissue-specific biomass/100)*tissue-specific C or N concentration), and secondly taking the sum of all tissue-specific C or N contents (leaves, stems, and roots), dividing the result by total biomass, and subsequently multiplying by 100. C:N ratios (both for the whole plant and for specific tissues) were calculated by dividing total and tissue-specific C concentrations by total and tissue-specific N concentrations, respectively.

### Statistical analyses

To test the effect of N treatment, tannin group, and genotype, and interactions thereof on plant growth, biomass allocation, and tissue chemical properties, we used general linear models with a partially nested and crossed design. In our model N treatment and tannin group served as main factors, and genotype served as a nested factor within tannin groups. All three factors were considered fixed, because they had been predetermined by us prior to setting up our experiment. We also included a blocking factor into our ANOVA model, but subsequently excluded it whenever it was not significant. Prior to analysis, data were tested for assumptions of normality and homoscedasticity, and were transformed as necessary to meet these assumptions. When a significant effect of N treatment, genotype, or of the genotype or tannin group by N treatment interaction was found, *post hoc* one-way ANOVAs and *post hoc* Student-Newman-Keuls (S-K-N) tests were performed to identify pairwise differences. All statistical analyses were performed using IBM^®^ SPSS^®^ statistics, version 21 (Armonk, NY, USA). All raw data are documented in reference material held at Umeå Plant Science Centre.

## Results

### Main effect of Nitrogen enrichment

Most growth and biomass allocation traits, as well as several tissue chemical properties responded positively to N addition, particularly to the high N treatment (Table 1 and S2 Fig). Contrary, some traits, including the root:shoot ratio, foliar condensed tannin concentrations, stem lignin, and all C:N ratios, decreased in response to N (Table 1 and S2 Fig). In some cases the reduction could only be detected when the plants had been grown at the highest N dose, as was the case for the root:shoot ratio, condensed tannin concentrations, and the C:N ratio in stems (S2 Fig). In contrast, stem lignin levels responded equally to both levels of added N, whereas distinct differences for all three N treatments could be found for the C:N ratio of leaves, roots, and the whole plant (S2 Fig).

**Table 1:** Main and interactive effects of nitrogen, genotype, and tannin group.

Responses	N_2_	GT(Tgr)_8_	Tgr_1_	GT(Tgr)N_16_	Tgr∙N_2_
**Growth and Allocation**					
Average Leaf Area (cm²)	**24.04** ***	**9.99** ***	2.77 ^0.098^	**1.83** *	**4.16** *
Total Leaf Area (cm²)	**188.46** ***	**6.14** ***	**7.36** **	1.57 ^0.083^	1.56 ^0.214^
RGR (cm day^-1^) ^b^	**362.05** ***	**34.13** ***	2.09 ^0.150^	**4.18** ***	**13.66** ***
Final Height (cm)	**140.32** ***	**31.72** ***	2.72 ^0.101^	**1.78** *	**11.84** ***
Leaf Biomass (g)	**245.96** ***	**4.95** ***	0.80 ^0.371^	**1.73** *	3.02 ^0.052^
Stem Biomass (g)	**198.10** ***	**4.55** ***	0.18 ^0.676^	1.70 ^0.053^	**8.25** ***
Root Biomass (g)	**34.91** ***	**4.57** ***	**17.40** ***	1.57 ^0.083^	2.03 ^0.135^
Root:Shoot Ratio ^b^	**198.01** ***	**48.14** ***	**53.11** ***	**2.57** **	2.84 ^0.062^
Biomass Increment (g day^-1^)	**233.71** ***	**2.11** *	2.87 ^0.092^	1.17 ^0.298^	**4.13** *
Total Biomass (g)	**133.73** ***	1.76 ^0.090^	**4.04** *	1.14 ^0.328^	**3.96** *
**Tissue Chemistry**					
Condensed Tannins (mg g^-1^ DW)	**103.41** ***	**16.04** ***	**114.31** ***	1.58 ^0.081^	**4.37** *
Lignin Total (%)	1.54 ^0.217^	0.43 ^0.903^	0.14 ^0.713^	1.15 ^0.312^	2.67 ^0.072^
Lignin Leaves (%) ^b^	1.62 ^0.201^	0.66 ^0.724^	0.09 ^0.764^	1.13 ^0.329^	0.05 ^0.956^
Lignin Stems (%)	**5.06** **	1.51 ^0.157^	0.69 ^0.408^	**2.24** **	0.68 ^0.508^
Lignin Roots (%) ^b^	0.35 ^0.706^	1.91 ^0.063^	1.67 ^0.199^	0.65 ^0.841^	2.04 ^0.133^
C Total (%)	2.80 ^0.064^	1.16 ^0.327^	0.00 ^0.992^	1.28 ^0.216^	0.66 ^0.520^
C Leaves (%)	**95.32** ***	**8.87** ***	**10.82** **	0.84 ^0.635^	0.49 ^0.616^
C Stems (%) ^b^	0.19 ^0.823^	1.49 ^0.167^	0.01 ^0.927^	1.68 ^0.057^	0.08 ^0.920^
C Roots (%)	0.68 ^0.508^	0.80 ^0.604^	0.26 ^0.611^	1.15 ^0.317^	0.47 ^0.625^
N Total (%) ^b^	**155.25** ***	1.04 ^0.407^	0.77 ^0.382^	1.09 ^0.366^	2.44 ^0.091^
N Leaves (%)	**486.01** ***	**10.57** ***	2.56 ^0.112^	**1.78** *	1.10 ^0.335^
N Stems (%)	**51.32** ***	0.72 ^0.675^	0.16 ^0.687^	1.19 ^0.286^	1.20 ^0.304^
N Roots (%)	**7.71** **	0.66 ^0.722^	1.15 ^0.285^	0.94 ^0.522^	1.27 ^0.285^
C:N Total ^b^	**208.20** ***	**2.07** *	0.57 ^0.453^	0.79 ^0.692^	**5.36** **
C:N Leaves	**227.09** ***	**8.67** ***	0.23 ^0.631^	1.23 ^0.253^	0.27 ^0.767^
C:N Stems	**10.64** ***	0.68 ^0.710^	0.03 ^0.858^	0.84 ^0.633^	0.88 ^0.417^
C:N Roots ^b^	**66.89** ***	1.58 ^0.136^	0.04 ^0.843^	1.07 ^0.384^	**3.79** *

ANOVA summary testing growth and tissue chemistry traits of *Populus tremula* plants in response to nitrogen addition (N), tannin group (Tgr), genotype nested within tannin group (GT(Tgr)), and interactions thereof. F-values are followed by significance levels:

***P<0.001,

**P<0.01,

*P<0.05.

Degrees of freedom (df) for the numerator are indicated in subscript. Denominator df were always 150, unless the block effect

^b^ was significant, and included into the analysis, which reduced the denominator df to 148.

### Main effect of genotype

The initial cross-nested ANOVAs suggested significant genotype effects for 14 traits (Table 1), but the *post hoc* one-way ANOVAs confirmed these GT effects only for nine traits (S3 Fig). Moreover, S-N-K *post hoc* tests showed pairwise differences for only seven of those traits (S3 Fig). Differences among genotypes for growth and biomass allocation responses were primarily observed for GT115 and GT60 (S3 Fig). Averaged across all N addition levels, GT23 had the smallest leaves, while GT50 and GT115 grew the largest leaves. Although GT115 possessed large leaves, this genotype showed the lowest daily height increment, and thus obtained the shortest final height. Genotype 115 expressed low above-ground growth, but invested largely into below-ground tissue. It had the highest root biomass and root:shoot ratio of all genotypes. In contrast, GT60 exhibited the highest daily height growth, and reached the tallest final height. Genotype 60, together with GT18 and GT5, had the lowest root:shoot ratio.

The chemistry traits foliar condensed tannin and foliar C concentrations were also influenced by genotype. Condensed tannin levels ranged from 26.4 ± 3.1 mg g^-1^ DW to 85.6± 6.6 mg g^-1^ DW in GT115 and GT65, respectively (S3 Fig). Both GT115 and GT60 expressed the lowest foliar condensed tannin concentrations. Foliar C concentrations were lowest in GT72 and GT115, and highest in GT18.

### Genotype responses to Nitrogen enrichment

**Fig 2:**
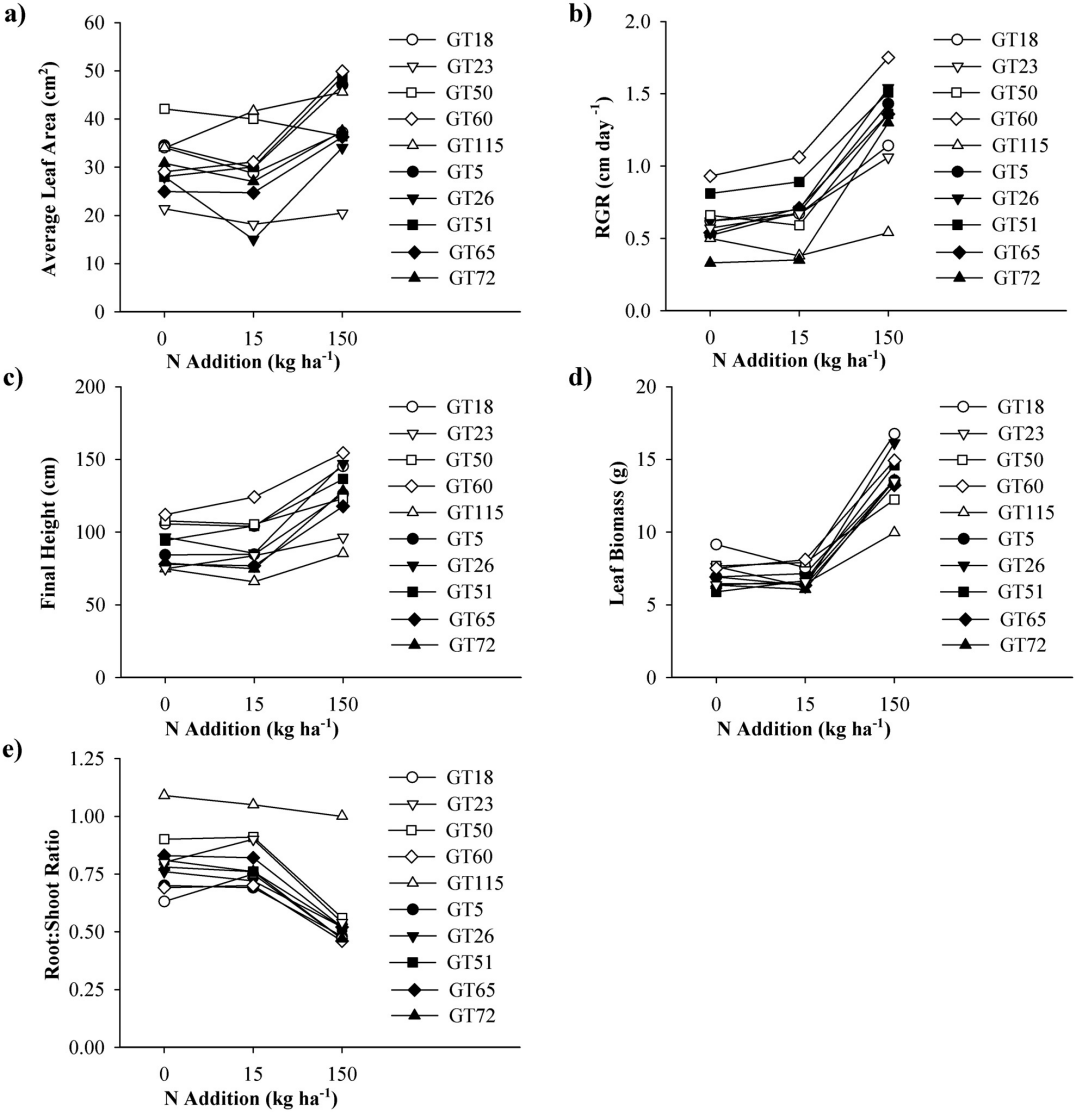
Growth traits of 10 aspen genotypes under 3 nitrogen conditions. Reaction norm plots showing plant growth traits for 10 aspen genotypes grown at three nitrogen addition levels: a) average leaf area, b) relative growth rate c) final height, d) leaf biomass, and e) root:shoot ratio. Data are means, and each line represents a single genotype.

Interactive effects of genotype (nested within tannin group) and N treatment were detected for several growth and biomass allocation response variables, including average leaf area, RGR, final height, leaf biomass, and the root:shoot ratio (Table 1). For each response trait, these interactive effects were due to different combinations of genotypes expressing high and low values under the different N addition levels (Fig 2 and S4 Fig). Average leaf area (Fig 2a and S4 Fig), for example, was highest in GT50 under ambient N conditions, whereas GT115 had the largest leaves at the low N level. Genotypes 60, 115, 5, and 51 all had equally large leaf areas at the highest N level. The lowest values for average leaf area were expressed by GTs 23, 60, 26, 51, and 65 under ambient N conditions, by GT23 and GT26 at the low N level, and by GT23 at the high N level. Relative growth rate for height (Fig 2b and S4 Fig) was highest in GT60 at all three N treatments. Genotype 72 was the slowest grower at the ambient and low N addition level. At the low N addition level GT72 overlapped in response with GT115 that grew slowest at the high N level. Genotype 60 always grew tallest (Fig 2c and S4 Fig), but could not be distinguished from GT18 and GT50 at the ambient N level. Slow-growers with low final height included GTs 23, 115, 5, 65 and 72 at ambient, GTs 115, 65 and 72 at low, and GT23 and GT115 at high N conditions, respectively.

Genotypic differences in leaf biomass were not apparent under ambient and low N conditions (Fig 2d and S4 Fig). However, at the high N addition level GT18 and GT26 had most leaf biomass, and GT115 least. Genotype 115 expressed the highest root:shoot ratio irrespective of N treatment, and always invested equally into below- and above-ground tissue (Fig 2e and S4 Fig). Under ambient N conditions, GT18 had the lowest root:shoot ratio, which shifted to GTs 60, 5 and 26 under low N addition. In the high N situation, all genotypes (apart from GT115) invested around twice as much into above-ground than into below-ground biomass.

**Fig 3:**
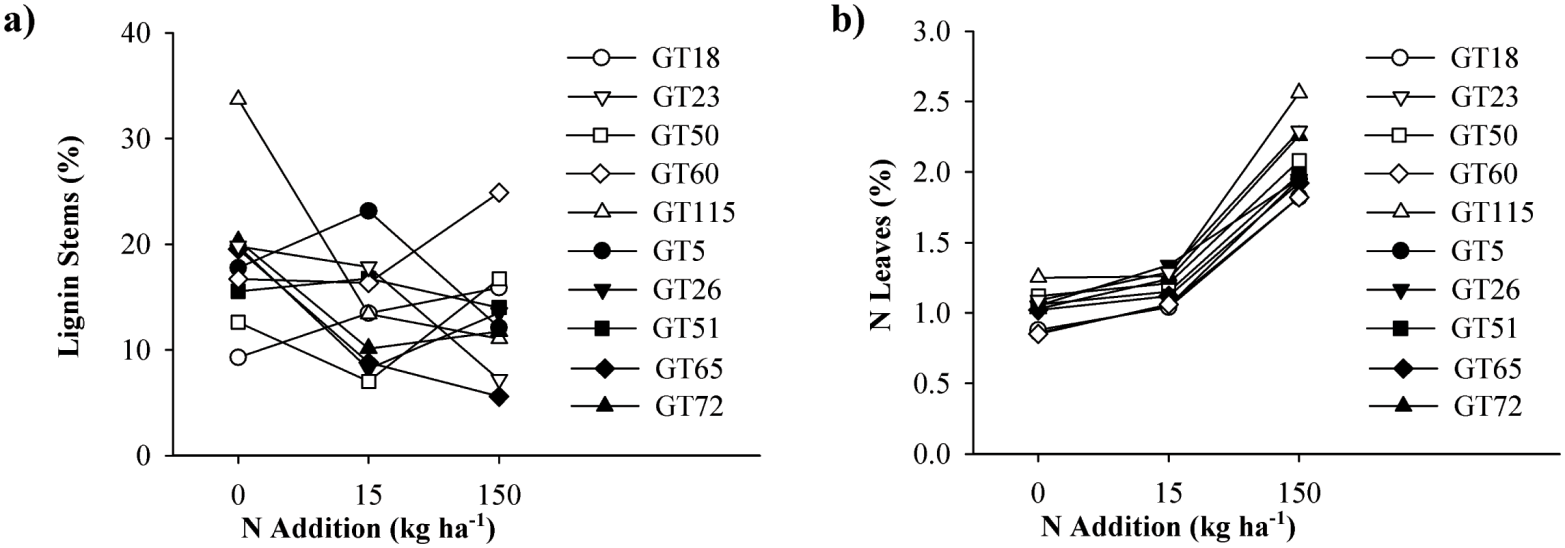
Chemistry traits of 10 aspen genotypes under 3 nitrogen conditions. Reaction norm plots showing tissue chemistry traits for 10 aspen genotypes grown at three nitrogen addition levels: a) stem lignin content, and b) leaf nitrogen content. Data are means, and each line represents a single genotype.

Interactive effects between genotype and N were also detected for two tissue chemistry variables, namely stem lignin and foliar N (Table 1). Stem lignin varied among genotypes in the control and high N situation, whereas no genotypic difference was found at the low N addition level (Fig 3a and S4 Fig). Under ambient N conditions GT115 had the highest stem lignin value (33.7%, which was almost double the amount found for most other GTs), whereas under high N addition GT60 expressed the highest stem lignin value (24.9%, and hence twice as much as most other GTs). The lowest stem lignin values were found for GT18 and GT50 under ambient, and for GT65 under high N conditions. Foliar N concentrations (Fig 3b and S4 Fig) were highest in GT115 under ambient and high N levels, and in GT26 under the low N level. Genotypes 18, 60 and 5 showed the lowest foliar N concentrations under ambient N, GTs 18 and 5 under low N, and GTs 18 and 60 under high N addition.

### Main effect of tannin group

Foliar condensed tannin levels differed between high- and low-tannin plants under ambient N conditions, confirming the successful establishment of the low- and high-tannin group in our experiment (independent sample t-test: t = 2.798; P 0.023; Fig 1b). Furthermore, averaged across the three N addition levels, the low-tannin group consistently expressed lower condensed tannin concentrations than the high-tannin group (Table 1; low Tgr: 40.94 ± 2.37 mg g^-1^ DW; high Tgr: 65.66 ± 3.16mg g^-1^DW).

Several other growth and biomass allocation traits (total leaf area, root biomass, root:shoot ratio, and total biomass), and one additional tissue chemistry trait (foliar C concentrations) significantly differed among tannin groups (Table 1). Plants in the low-tannin group showed a larger total leaf area (low Tgr: 1538.29 ± 62.13 cm^2^; high Tgr: 1394.93 ± 65.36 cm^2^), had a greater root mass (low Tgr: 14.58 ± .46 g; high Tgr: 12.47 ± 0.45 g), had a higher root:shoot ratio (low Tgr: 0.76 ± 0.02; high Tgr: 0.68 ± 0.02), and produced more biomass at harvest (low Tgr: 34.89 ± 1.17 g; high Tgr: 32.33 ± 1.44 g) than plants in the high-tannin group. Moreover, foliar C concentrations were elevated in the low-tannin group compared to the high-tannin group (low Tgr: 47.84 ± 0.12%; high Tgr: 47.48 ± 0.12%).

### Tannin group response to N enrichment

**Fig 4:**
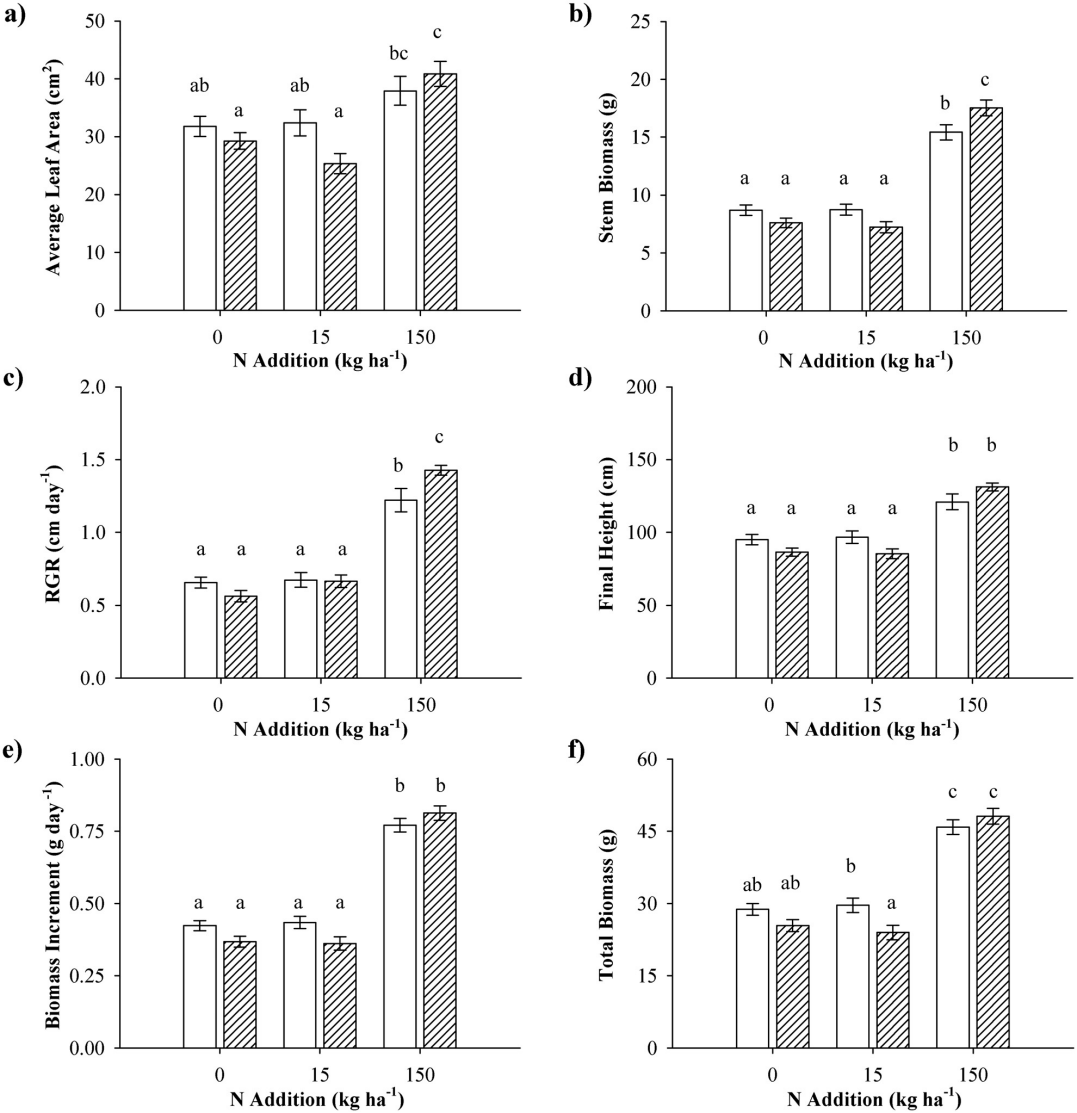
Growth traits for low and high tannin aspens under 3 nitrogen conditions. Plant growth traits for aspen genotypes expressing low (white) and high (hatched) intrinsic levels of foliar condensed tannins grown at three nitrogen addition levels: a) average leaf area, b) stem biomass, c) relative growth rate, d) final height, e) daily biomass increment, and f) total biomass. Data are means (± SE) of pooled data. Different letters indicate differences according to the Student-Newman-Keuls *post hoc* test.

The interaction between tannin group and N treatment affected some growth and biomass allocation traits including average leaf area, RGR, final height, stem biomass, biomass increment, and total biomass (Table 1). For all these growth traits, a pattern of high-tannin plants expressing slightly smaller values than low-tannin plants at ambient and low N conditions, and slightly higher values at the high N addition level could be observed (Fig 4a–4f). Although this pattern was repeated for all growth traits, for three of the traits (average leaf area, final height, and biomass increment; Fig 4a, 4d and 4e), *post-hoc* analyses could not identify the source of the tannin group x N treatment effect. However, for stem biomass (Fig 4b) and RGR (Fig 4c), *post hoc* analysis revealed that the significant tannin group by nitrogen interaction was due to differences between the tannin groups under the high N treatment, whereas for total biomass (Fig 4f) it was due to difference between tannin groups at the low N addition level.

**Fig 5:**
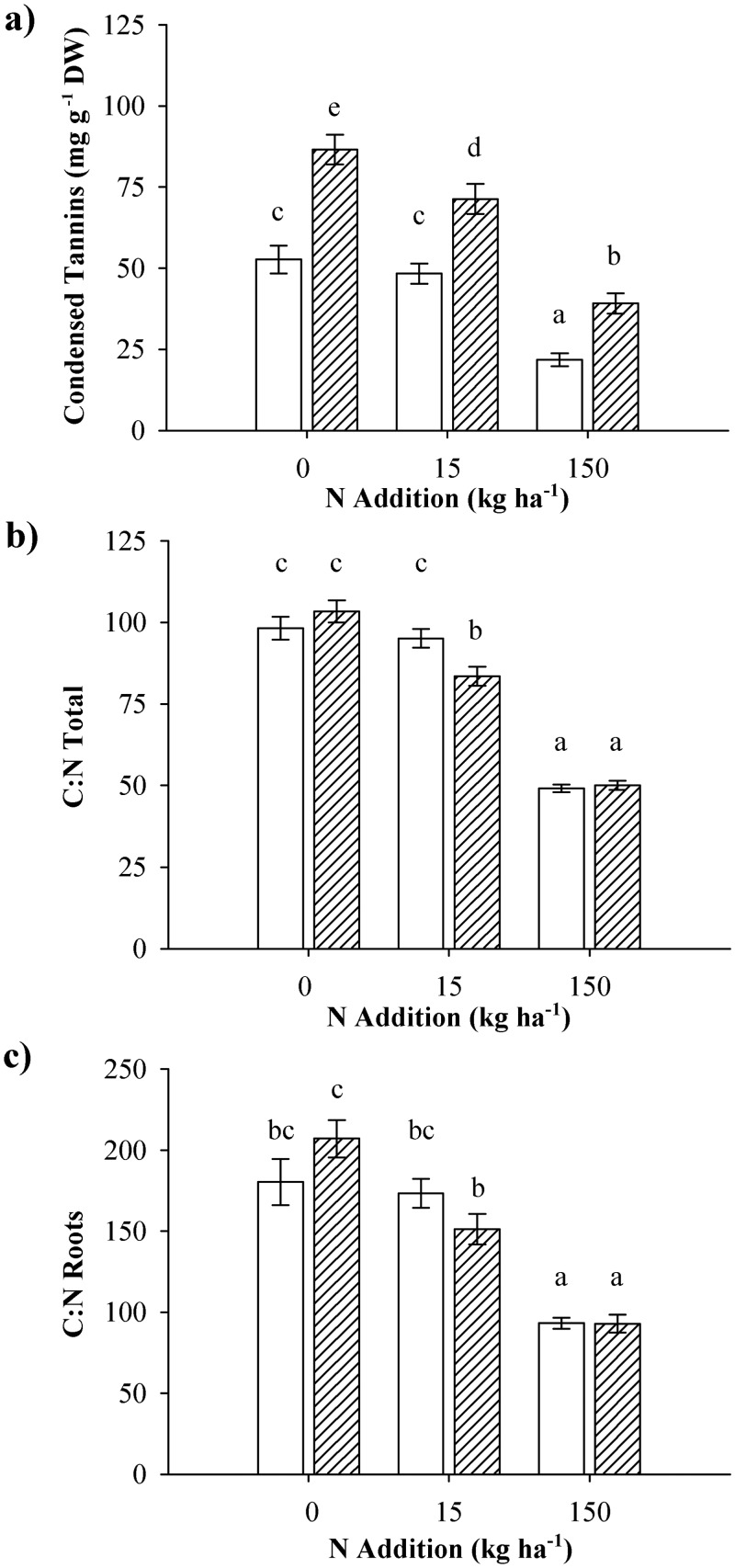
Chemistry traits for low and high tannin aspens under 3 nitrogen conditions. Tissue chemistry traits for aspen genotypes expressing low (white) and high (hatched) intrinsic levels of foliar condensed tannins grown at three nitrogen addition levels: a) foliar condensed tannins, b) C:N ratio at the plant level, and c) root C:N ratios. Data are means (±SE) of pooled data. Different letters indicate differences according to the Student-Newman-Keuls *post hoc* test.

Plant tissue chemistry traits with interactive responses to tannin group and nitrogen addition included foliar condensed tannin concentrations, and C:N ratios of roots and the entire plant (Table 1). The greatest difference in foliar condensed tannin concentrations was expressed in the control treatment, with less extreme differences occurring in response to the other two N addition treatments (Fig 5a). All plants expressed lower tannin levels in response to N addition, but high-tannin plants were more sensitive, and responded already to the low N treatment, whereas low-tannin plants first showed decreased condensed tannin concentrations when grown under the highest N addition level. The C:N ratio of both tannin groups was highest in the ambient situation, in which no differentiation between tannin groups was apparent (Fig 5b). The C:N ratio of the high-tannin plants became significantly lower than the C:N ratio of the low-tannin plants in response to the low N treatment, whereas no difference between tannin groups was present in response to the high N treatment. For the root C:N ratio (Fig 5c), *post hoc* analysis could not clearly indicate the source of the interactive effect of tannin group and N treatment. Tannin group differences for the root C:N ratio were highest in the ambient N situation, in which high-tannin plants showed a higher root C:N ratio than low-tannin plants. At the low N addition level, low-tannin plants expressed a higher root C:N ratio than high-tannin plants, whereas under the high N addition level no difference between tannin groups occurred.

## Discussion

We found that all growth and biomass allocation variables, as well as many tissue chemistry variables responded to N, which shows that we successfully established a N gradient that impacted plant growth, such as exists within northern latitudes [1, 2]. Plant responses to N were often absent for the N dose simulating atmospheric deposition, but present for the N dose mimicking forest fertilization. Nitrogen addition caused plants to grow more, while producing tissue with lower concentrations of condensed tannins and lignin (stem tissue only). An overall negative relationship between foliar condensed tannins and plant growth indicates a trade-off between growth and defense [17]. Indeed, we found that growth increased, and condensed tannins decreased with N addition, which supports plant defense hypotheses, and confirms results from several other studies [23, 33, 34, 54]. In addition to main N effects, we also observed main GT effects for many growth and biomass allocation variables, and some chemistry traits. All growth and biomass allocation responses, except total biomass, differed among GTs, which is consistent with observations by Randriamanana et al. [28]. Genotypes also showed differences in tissue chemistry variables, particularly for leaves. Foliar condensed tannin concentrations varied substantially among GTs, which agrees with studies of *P*. *tremula* [28, 36], and *P*. *tremuloides* (e.g. [26], [55]).

Significant interactive effects between genotype and N were found for several traits, most strongly for relative growth rate, root:shoot ratio, and stem lignin concentrations. These interactions often occurred because individuals within the population responded idiosyncratically to differences in N availability, as also shown for *P*. *tremuloides* [11, 15, 23, 34], and species of *Salix* [22]. Plant development is controlled by a large number of genes that are randomly inherited [36, 43, 45, 56]. For specific traits that are strongly linked to N uptake this randomness may be expressed in the priority of functions (e.g. allocation priority between growth and defense) or in biosynthetic precedence of specific chemical pools (e.g. lignins), resulting in traits that are poorly correlated among genotypes [36, 43].

We found tannin group differences for nearly half of the studied growth and biomass allocation traits, and for two tissue chemistry traits. Low-tannin plants produced a greater total leaf area, a higher total biomass, and had higher foliar C concentrations indicating that growth vs. defense trade-offs for a given species are not only plastic, but are to some extend also genetically controlled. Moreover, we observed significant tannin group x N treatment interactions for some growth and biomass allocation variables, and for three tissue chemistry responses. For total biomass and average leaf area, these interactive responses appeared to be driven by higher values in low-tannin plants under ambient or low N conditions, and a convergence of the high- and low-tannin group under high N conditions. This suggests that genetic control of tannin production may constrain total plant growth and leaf area, which is consistent with several plant defense hypotheses. However, our data also showed that plant plastic responses to N can eliminated this constraint, as low- and high-tannin groups converged to equal growth values when supplied with high levels of N. Furthermore, for stem biomass and relative height growth, high-tannin plants responded more positively to high N addition relative to low-tannin plants. Our data thus provide very weak evidence that genetic control on plant tannin production is a major constraint for plants in their response to gradients of N availability.

Plant intraspecific variability is proposed as a key mechanism by which plants are able to adapt to environmental change [15, 16, 57]. While many studies have identified the importance of phenotypic plasticity or genotypic variability in determining how plant populations respond to environmental change, our study was able to isolate how plastic responses differed among contrasting plant genotypes and genotype groups, thus providing several insights into understanding how plants respond to environmental change. For the low N treatment, which simulated an annual dose of maximum N deposition rates experienced in the boreal region, and for which many plant traits were unresponsive, we observed that high-tannin plants grew significantly less than low-tannin plants (Fig 4f). This suggests that within this relatively low N context, the production of tannins has a negative cost that impacts growth. While this trade-off favored low-tannin plants in our experimental setting, genotypes with high tannin levels may gain greater benefit in natural environments, where damage by pathogens and herbivores is likely to occur [55], ([58] and references therein).

In contrast to the low N addition treatment, there was no apparent growth cost for high-tannin plants subjected to high N addition rates, which simulated industrial forest fertilization rates. Instead, for most growth traits high-tannin plants performed at least as well compared to low-tannin producers, when they received the highest N dose. This suggests that aspen trees that have a genetically controlled high baseline production of tannins are potentially associated with a wider reaction norm compared to those that produce lower levels of foliar condensed tannins, at least at the highest rate of N addition used in our experiment. Thus, our results could suggest that high-tannin producers may express a higher growth plasticity and thus utilize large doses of N more efficiently. This insight has several implications. Firstly, the steeper reaction norm of growth responses in high-tannin genotypes suggests a higher plasticity to nitrogen addition, and potentially an advantage when adapting to higher concentrations of soil nitrogen. Secondly, models of optimal allocation should not only follow nutrient availability (such as in [18, 19]), but also the intrinsic capacity of a plant to balance carbon needs for growth and defense compounds. Thirdly, despite the importance of tannin groups in determining how plants responded to the two N addition levels, it is worth noting that many individual genotypes responded differently to the low N level, regardless of their tannin levels. These idiosyncratic responses to nutrients highlight that many other genetic factors are likely important in regulating plant responses. While the effect of tannin group may add little explanation to the allocation differences observed for young aspen plants in our experiment, it may have importance in a longer time perspective, under field conditions, under which the plants are exposed to multiple stresses.

## Supporting Information

S1 Data Raw data sheet for Bandau et al. study. The complete set of raw data collected for the study: “Genotypic tannin levels in *Populus tremula* impact the way nitrogen enrichment affects growth and allocation responses for some traits and not for others”.
(XLSX)

S1 Fig Tannin concentrations in selected aspen genotypes from the SwAsp collection. Condensed tannin (CT) concentrations in foliage of *Populus tremula* trees from the SwAsp collection (for details see the method section and references therein). Sampling years were 2008 for the Ekebo garden, and 2008 and 2009 for the Sävar garden. Only data for the 10 genotypes (GTs) selected for this study are displayed. For each GT up to 6 replicates were analyzed. Further displayed are mean condensed tannin concentrations for each GT in each garden during each sampling year (data aggregated by replicates—annotated as “mean for reps”), mean condensed tannin concentrations for each GT in each garden (means further aggregated by year—annotated as “mean for year”), mean condensed tannin concentration for each GT (means further aggregated by GT—annotated as “mean for GT”), and mean condensed tannin concentration for each tannin group (Tgr) (means further aggregated by tannin group—annotated as “mean for Tgr”).
(EPS)

S2 Fig Effects of nitrogen on growth and tissue chemistry traits. Growth and tissue chemistry traits for which the cross-nested ANOVAs (Table 1) indicated significant effects of Nitrogen treatment. F-values and indication of significance (*** P < 0.001, ** P < 0.01) of one-way ANOVAs testing the single effect of N addition. Means ± SE are given for individual N levels. *Post hoc* letters refer to differences between means as determined by S-N-K *post hoc* tests. Different letters indicate significantly different means. Units are given in Table 1.
(EPS)

S3 Fig Effects of genotype on growth and tissue chemistry traits. Growth and tissue chemistry traits for which the cross-nested ANOVAs (Table 1) indicated significant effects of GT(Tgr). F-values and indication of significance (*** P < 0.001, * P < 0.05) of one-way ANOVAs testing the single effect of genotype. Means ± SE are given for individual genotypes. *Post hoc* letters refer to differences between means as determined by S-N-K *post hoc* tests. Different letters indicate significantly different means. Units are given in Table 1.
(EPS)

S4 Fig Interactive effects of nitrogen and genotype on growth and tissue chemistry traits. Growth and tissue chemistry traits for which the cross-nested ANOVAs (Table 1) indicated significant effects of the GT(Tgr) x N interaction. Means ± SE are given for the individual genotypes (GT) (sorted by tannin group) within individual N addition levels. *Post hoc* letters refer to differences between means as determined by S-N-K *post hoc* tests. Different letters indicate significantly different means. Units given in Table 1.
(EPS)
